# Limited Systemic Sclerosis Patients with Pulmonary Arterial Hypertension Show Biomarkers of Inflammation and Vascular Injury

**DOI:** 10.1371/journal.pone.0012106

**Published:** 2010-08-17

**Authors:** Sarah A. Pendergrass, Everett Hayes, Giuseppina Farina, Raphael Lemaire, Harrison W. Farber, Michael L. Whitfield, Robert Lafyatis

**Affiliations:** 1 Department of Genetics, Dartmouth Medical School, Hanover, New Hampshire, United States of America; 2 Rheumatology Section, Arthritis Center, Boston University School of Medicine, Boston, Massachusetts, United States of America; 3 Pulmonary Section, Boston University School of Medicine, Boston, Massachusetts, United States of America; University of Giessen Lung Center, Germany

## Abstract

**Background:**

Pulmonary arterial hypertension (PAH) is a common complication for individuals with limited systemic sclerosis (lSSc). The identification and characterization of biomarkers for lSSc-PAH should lead to less invasive screening, a better understanding of pathogenesis, and improved treatment.

**Methods and Findings:**

Forty-nine PBMC samples were obtained from 21 lSSc subjects without PAH (lSSc-noPAH), 15 lSSc subjects with PAH (lSSc-PAH), and 10 healthy controls; three subjects provided PBMCs one year later. Genome-wide gene expression was measured for each sample. The levels of 89 cytokines were measured in serum from a subset of subjects by Multi-Analyte Profiling (MAP) immunoassays. Gene expression clearly distinguished lSSc samples from healthy controls, and separated lSSc-PAH from lSSc-NoPAH patients. Real-time quantitative PCR confirmed increased expression of 9 genes (ICAM1, IFNGR1, IL1B, IL13Ra1, JAK2, AIF1, CCR1, ALAS2, TIMP2) in lSSc-PAH patients. Increased circulating cytokine levels of inflammatory mediators such as TNF-alpha, IL1-beta, ICAM-1, and IL-6, and markers of vascular injury such as VCAM-1, VEGF, and von Willebrand Factor were found in lSSc-PAH subjects.

**Conclusions and Significance:**

The gene expression and cytokine profiles of lSSc-PAH patients suggest the presence of activated monocytes, and show markers of vascular injury and inflammation. These genes and factors could serve as biomarkers of PAH involvement in lSSc.

## Introduction

Pulmonary arterial hypertension (PAH) is a common complication of systemic sclerosis (SSc) associated with high mortality despite modest improvements in survival due to increased screening and treatment. PAH occurs more frequently in limited SSc (lSSc) than in diffuse SSc (dSSc) [1]. Early detection and treatment of PAH secondary to SSc (SSc-PAH) might lead to better patient outcome [2]. For example, early treatment of renovascular disease in SSc patients results in improved renal outcomes [3]. In addition, hypoxia from progressive PAH may accelerate vascular injury by stimulating increased ET-1, VEGF, PDGF and endothelial apoptosis [4], [5]. Current tools used in screening for PAH in SSc patients include echocardiography, pulmonary function testing, and levels of B-type natriuretic peptide (BNP), none of which have demonstrated adequate sensitivity or specificity [2]. Thus, SSc patients could benefit from biomarkers that permit earlier detection of patients at risk for PAH.

SSc patients can have severe disease in several different vascular beds, resulting in digital ischemia, telangiectasias, scleroderma renal crisis and PAH. Similar to other vascular pathology in SSc, SSc-PAH vascular remodeling consists of intimal thickening of pulmonary arterioles and capillaries due to intimal cell proliferation and deposition of extracellular matrix [6]. Inflammation may play a role in SSc-PAH, as patients have circulating autoantibodies and perivascular inflammatory cell infiltrates such as T- and B-lymphocytes, and macrophages [6]. While there are similarities in the histological appearance of vascular lesions and the presumed pathogenesis between idiopathic PAH (IPAH) and SSc-PAH, the risk of death for SSc-PAH patients is higher [7], [8]. There are also indications that SSc-PAH patients have fewer plexiform lesions and more intimal hyperplasia [6], [9], as well as differences in the involvement of the pulmonary veins [6].

Since pulmonary tissue is not readily accessible, it is difficult to perform gene expression studies in SSc-PAH analogous to those of SSc skin biopsies [10], [11], [12]. An alternative is to analyze gene expression of peripheral blood mononuclear cells (PBMCs). Two studies have investigated the gene expression of PBMCs in IPAH and SSc-PAH. Bull et al. examined PBMC samples from 7 patients with IPAH, 3 patients with SSc-PAH (in a total of 8 patients with PAH related to a secondary cause), and 6 healthy controls [13]. Genes were identified that discriminated PAH patients from healthy controls. Grigoryev et al. analyzed PBMCs from 9 patients with IPAH, 10 patients with SSc-PAH, and 5 healthy controls [14]. Gene expression concordant between the IPAH and SSc-PAH groups was contrasted with discordant gene expression. Neither study examined the alterations in PBMC gene expression specifically attributable to PAH in SSc. We hypothesized that PBMC gene expression would specifically separate lSSc patients from healthy controls, as well as SSc patients with and without PAH. We report here PBMC gene expression defining lSSc patients with and without PAH, and healthy controls.

## Results

Although PAH can occur in patients with dSSc, in order to provide a more homogeneous population for analysis, we limited this study to patients with lSSc, where it occurs more commonly. Gene expression of PBMC samples was analyzed for 21 lSSc patients without PAH (lSSc-NoPAH), 15 lSSc patients with PAH (lSSc-PAH), and 10 healthy controls. For three patients (two lSSc-NoPAH and one lSSc-PAH), an additional PBMC sample was analyzed one year after the baseline, resulting in 49 total samples. With 5 technical replicates, a total of 54 microarrays were analyzed. LSSc patients with mildly elevated pulmonary capillary wedge pressure (PCWP) (>15 to ≤18) were included in our primary analyses consistent with the REVEAL registry with similar rationale [15]. Patients that had a mild increase in PCWP included in the primary analysis all had significantly elevated pulmonary vascular resistance (PVR, see Table 1), and significant increases in both the pulmonary artery diastolic minus pulmonary capillary wedge pressure (PAd-PCWP) gradient (>10) and the transpulmonary gradient (>15). Thus, each was considered to have PAH by the pulmonary hypertension expert caring for the patient. Further data supported the diagnoses of PAH in these patients, Patient 66 had a right heart catheterization two months prior to the catheterization carried out on the day of study enrollment, showing similar elevated pressures (mPAP = 49) but a normal PCWP (11). Patient 31 had relatively mild PAH (mPAP = 32) and a relatively mild increase in PVR (207), but no evidence of right (RV) or left ventricular (LV) dysfunction on echocardiogram or by cardiac output. Patient 42 had severely elevated mPAP and PVR, with an echocardiogram showing normal LV function but severely dilated RV consistent with severe PAH. Patient 89 had relatively mild PAH (mPAP = 37, PVR = 205), with mild elevation of the PCWP (PCWP = 16), but a PAd 10 mmHg greater than the PCWP consistent with PAH. A limited number of patients with pulmonary fibrosis, some with extensive pulmonary fibrosis (2 in each group of lSSc-PAH and lSSc-noPAH patients), were also included in our primary analyses because PAH is common in these SSc patients [16] and it was deemed important to understand whether biomarkers for lSSc-PAH patients with pulmonary fibrosis were similar to biomarkers for lSSc-PAH patients without pulmonary fibrosis. To further support the results of our primary analysis which included these patients, we also carried out a secondary analysis excluding patients with elevated PCWP and/or extensive pulmonary fibrosis, described below.

**Table 1:** Clinical and hemodynamic data on subjects associated with PBMC sample arrays.

Subject Identifier	Age	Gender	ILD	Medications	mPAP (mm Hg)	PCWP (mm Hg)	PVR (dyn.s/cm^−5^)	CO/CI (L/min/L/min/M^2^)	FVC (% predicted)	DLCO (% predicted)
LSSC_PAH_Pat02	67	F	none	tadalafil	31	14	282	5.1/3.3	79	58
LSSC_PAH_Pat29	62	M	none	epoprostenol	58	14	475	7.4/4.8	80	40
LSSC_PAH_Pat31	70	F	none	none	32	16	207	5.8/3.2	100	68
LSSC_PAH_Pat42	59	F	mild ILD	none	57	18	745	4.4/2.5		
LSSC_PAH_Pat45	52	F	none	none	52	11	625	5.5/3.4		48
LSSC_PAH_Pat48	63	M	mild ILD	bosentan, sildenafil	45	10	451	6.2/3.2	75	33
LSSC_PAH_Pat54	65	F	none	none	34	8	378	5.5/3.0		
LSSC_PAH_Pat60	70	F	none	none	48	9	706	4.3/2.4	94	50
LSSC_PAH_Pat60_1yr	71	F	none	sildenafil					87	47
LSSC_PAH_Pat62	70	F	none	epoprostenol	43	8	823	3.3/1.8	86	44
LSSC_PAH_Pat64	64	F	extensive ILD	none	33	13	222	5.4/2.9	59	54
LSSC_PAH_Pat66	80	F	none	sildenafil	52	16	846	3.4/2.0		
LSSC_PAH_Pat83	64	F	none	none	37	11	519	4.1/2.3	101	34
LSSC_PAH_Pat85	69	**M**	none	sildenafil	53	9	720		61	43
LSSC_PAH_Pat89	67	F	extensive ILD	none	37	16	205	8.2/2.6	48	43
LSSC_PAH_Pat90	56	F	mild ILD	epoprostenol	42	5	503	6.2/3.9	99	38
LSSC_NoPAH_Pat12	33	F	none	nifedipine					116	109
LSSC_NoPAH_Pat19	36	F	none	none					97	61
LSSC_NoPAH_Pat22	45	M	extensive ILD	nifidipine	21	10	109	8.1/5.7	54	29
LSSC_NoPAH_Pat35	46	F	none	none						
LSSC_NoPAH_Pat35_1yr	48	F	none	sildenafil					65	
LSSC_NoPAH_Pat47	69	F	none	benazepril, procardia					96	76
LSSC_NoPAH_Pat52	61	F	mild ILD	nifedipine						69
LSSC_NoPAH_Pat52_1yr	62	F	mild ILD	nifedipine						52
LSSC_NoPAH_Pat55	60	F	none	nitroglycerin cream					96	61
LSSC_NoPAH_Pat57	48	M	none	nifedipine	15	9	87	5.5/2.4	75	91
LSSC_NoPAH_Pat58	44	F	none	none	18	10	114	5.6/3.7	110	95
LSSC_NoPAH_Pat67	45	F	none	none					108	94
LSSC_NoPAH_Pat75	55	F	none	nifedipine					90	98
LSSC_NoPAH_Pat80	51	F	none	propanolol					105	82
LSSC_NoPAH_Pat81	37	F	none	nifedipine, nitropaste, trental						
LSSC_NoPAH_Pat82	76	F	none	metoprolol	21	13	125	5.1/3.0	96	51
LSSC_NoPAH_Pat86	51	F	extensive ILD	none					42	61
LSSC_NoPAH_Pat87	38	M	none	none					95	90
LSSC_NoPAH_Pat91	56	F	mild ILD	atenolol					117	65
LSSC_NoPAH_Pat98	56	F	none	nifedipine					89	82
LSSC_NoPAH_Pat100	39	F	none	nifedipine						
LSSC_NoPAH_Pat102	43	F	none	none					107	83
LSSC_NoPAH_Pat116	57	F	none	none					100	66

**Norml control patients were as follows:** Normal_NoPAH_Pat13 (F), Normal_NoPAH_Pat19 (M), Normal_NoPAH_Pat96 (M), Normal_NoPAH_Pat97 (M), Norma_NoPAH_pat106 (M), Normal_NoPAH_Pat108 (M), Normal_NoPAH_Pat111 (F), Normal_NoPAH_Pat117 (M), Normal_NoPAH_Pat118 (M).

### PBMC gene expression distinguishes lSSc and healthy controls

**Figure 1:**
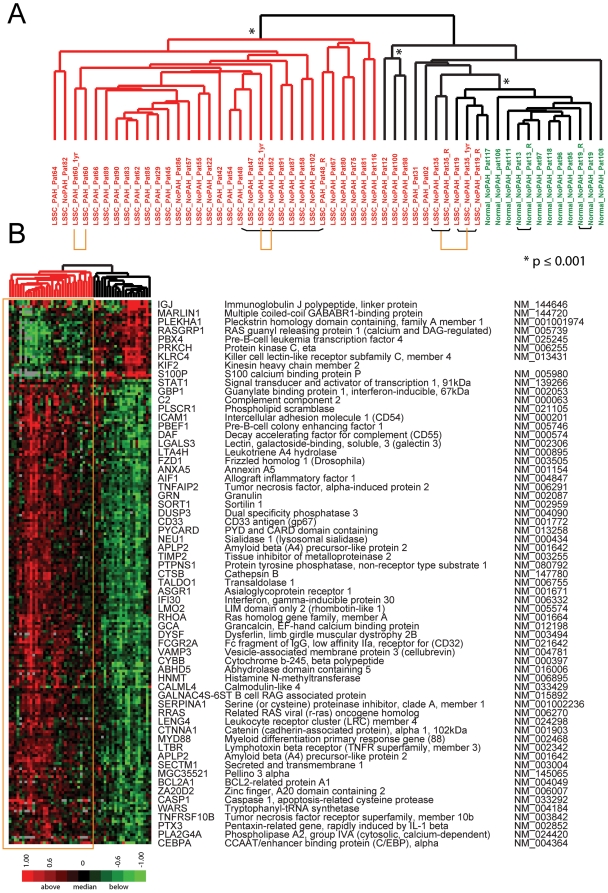
Gene expression differentiates lSSc PBMC samples from healthy controls. A. Clustering dendrogram using 206 probes that most differentiated lSSc (red identifiers) and healthy control samples (green identifiers; FDR cutoff<0.18%). Black bars beneath the sample identifiers connect technical replicates. Samples collected one year apart are indicated by yellow bars. Asterisks indicate statistically significant clustering determined by SigClust. The red dendrogram branch contains the majority of lSSc samples, and the black branch contains those lSSc samples most like healthy controls. B. Heat map showing expression of the 206 probes after 2-dimensional hierarchical clustering. Expression values above the mean for each probe are indicated in red and below the mean are indicated in green. The yellow box highlights the gene expression data for the dendrogram branch containing the majority of lSSc samples. A subset of genes is listed next to the heat map. The full figure with all probe names is available as Figure S1.

Data were first analyzed for genes that differentiated lSSc from healthy controls. The most significantly differentially expressed genes between lSSc patients, regardless of PAH status, and healthy controls were identified with Significance Analysis of Microarrays (SAM) [17]. 206 probes were selected with a false discovery rate (FDR) <0.18% and clustered hierarchically by both sample and probe (Figure 1). The division between the lSSc and healthy control samples was clear, with all healthy controls clustering together (Figure 1A). Four out of five technical replicates clustered either immediately adjacent to one another or with a single sample separating them (black bars, Figure 1A). Notably, all three PBMC samples collected one year after baseline demonstrated gene expression nearly identical to the initial samples (yellow bars, Figure 1A). Thus, for these three patients, the gene expression was stable over a period of one year, results remarkably consistent with the longitudinal analysis of gene expression in SSc skin (Pendergrass, Lafyatis, Whitfield, *In preparation*). The full figure with all probe names is available as Figure S1; the complete data file is available as Supplementary Data File S1.

The major dendrogram bifurcation places all healthy controls onto one branch, and divides the lSSc patients into two groups, despite selecting for genes that ideally stratify the groups (Figure 1). Statistically significant dendrogram branches (*, p≤0.001) were determined with SigClust [18]. The left branch is significant at the 0.001 level (red branches, Figure 1A) and includes PBMCs from 29 of 36 lSSc patients, while the remaining 7 lSSc samples group with healthy controls (black branches, Figure 1A). As observed for dSSc skin [10], most lSSc patients show an expression profile distinct from healthy controls (Figure 1B). The 7 lSSc patients that group with healthy controls therefore constitute a ‘normal-like’ group, similar to that observed in SSc skin [10].

Genes with increased expression in lSSc PBMCs are associated with inflammation and vascular injury. Those associated with inflammation include interleukin-1, interferon, and TNF-alpha regulated genes such as intracellular adhesion molecule 1 (ICAM1) [19], caspase 1 (CASP1) [20], lymphotoxin beta receptor TNFR superfamily, member 3 (LTBR) [21], signal transducer and activator of transcription 1 (STAT1), and allograft inflammatory factor (AIF-1) [22]. Genes associated with angiogenesis, proliferation, hypoxia and vascular injury include Pre-B-cell colony enhancing factor (PBEF1/Visfatin) [23], Guanylate binding protein 1 (GBP-1) [24], Tryptophanyl-tRNA synthetase (WARS) [25], Tissue inhibitor of metalloproteinase 2 (TIMP2), and CyBB cytochrome b-245 beta polpypetide (CYBB/NOX2) [26]. Genes with decreased expression in lSSc did not show cohesive biological processes but included protein kinase C eta (PRKCH), which had decreased expression in PBMCs from rheumatoid arthritis patients [27].

### Gene expression in lSSc-PAH

**Figure 2:**
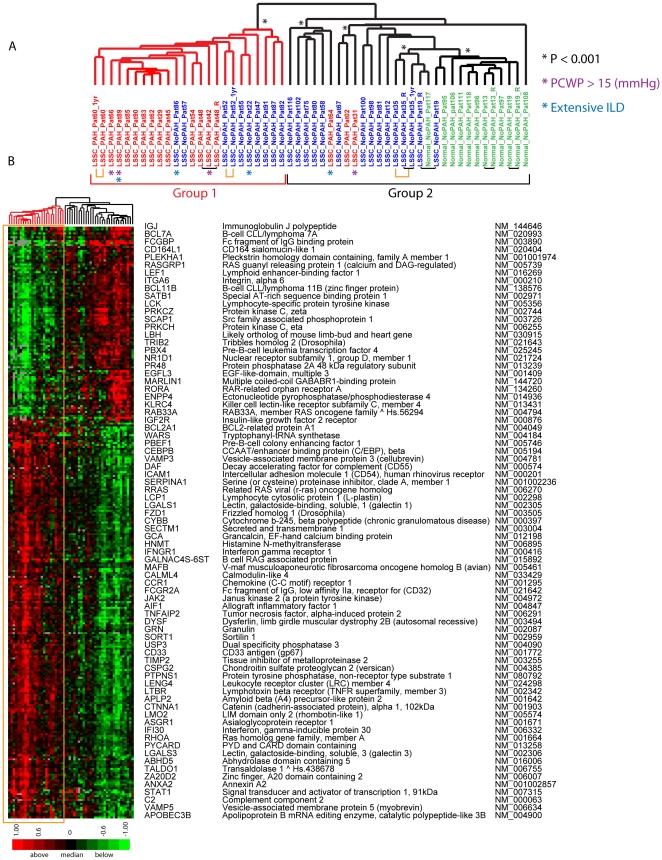
Gene expression differentiating lSSc-PAH, lSSc-noPAH and healthy controls. A. Hierarchical clustering dendrogram generated using 305 probes selected by a multi-group SAM analysis (FDR<0.14%). LSSc-PAH sample identifiers are indicated in red, lSSc-NoPAH in blue, and healthy controls in green. Black bars connect technical replicates and yellow bars connect samples collected one year apart. Statistically significant branches determined by SigClust are indicated. The major bifurcation in the dendrogram divides Group 1 and Group 2. B. Heat map showing the expression values of the 305 probes after 2-dimensional hierarchical clustering. Gene expression ratios are colored as in Figure 1. The yellow box highlights the gene expression of Group 1, which contains most of the lSSc-PAH PBMC samples. A subset of genes from the 305 probes are listed. The full figure with all probe names is available as Supplementary Figure S2. Patients with PCWP >15 (mm Hg) are indicated with a purple asterisk (*) and those with extensive ILD are indicated with a blue asterisk (*). The analysis was repeated without these patients and nearly identical groupings were obtained (Figure S3).

In addition to the gene expression differences found between the lSSc and healthy control samples, a multi-class SAM analysis identified gene expression that ideally distinguished lSSc-PAH, lSSc-NoPAH, and healthy controls. A total of 305 probes were selected with an FDR<0.14%, and clustered hierarchically in the gene and sample dimensions (Figure 2). Twelve of 15 lSSc-PAH samples clustered together on a significant branch (Group 1, Figure 2A), and showed the largest differences in gene expression relative to controls. Nine of 21 lSSc-NoPAH samples clustered on the same branch and showed intermediate expression levels. Three lSSc-PAH samples (patients 64, 31, and 2) grouped on the opposite branch (Group 2, Figure 2A) with lSSc-NoPAH samples. All technical replicates clustered adjacent to each other, or with only a single sample separating them. Samples collected one year later showed gene expression most similar to the previous sample. The full figure with all probe names is available as Figure S2; the data file is available as Supplementary Data File S2.

**Figure 3:**
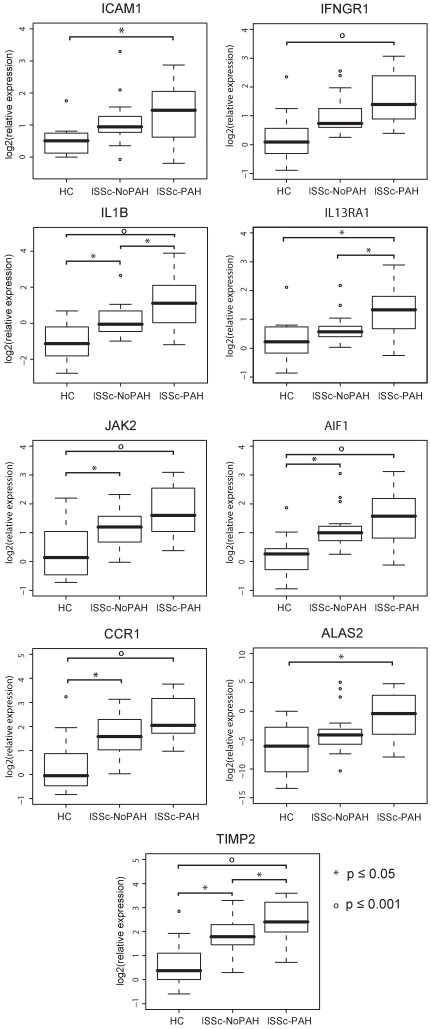
Quantitative Real Time PCR validation of gene expression. RT-PCR validation was performed for nine genes (ICAM1, IFNγR1, IL1B, IL13Rα1, JAK2, AIF1, CCR1, ALAS2, TIMP2) in healthy control, lSSc-NoPAH, and lSSc-PAH samples. Bars indicate comparisons with statistically significant differential expression. Symbols indicate the level of significance between groups (asterisks p≤0.05, open circles p≤0.001).

A striking feature of the gene expression is a gradient with the largest differences between the lSSc-PAH and controls, with lSSc-NoPAH showing intermediate expression levels. To validate this finding, the most significant differentially expressed genes were analyzed by quantitative RT-PCR. Nine genes were validated (Figure 3): intercellular adhesion molecule 1 (ICAM1), associated with vascular injury; Interferon-γ receptor 1 (IFNγR1), interleukin 1 beta (IL1B), interleukin 13 receptor α1 (IL13Rα1), janus kinase 2 (JAK2), allograft inflammatory factor 1 (AIF1) and chemokine (C-C motif) receptor 1 (CCR1), all of immunological relevance; Aminolevulinate delta synthase 2 (ALAS2), a possible regulator of the response to hypoxia; and tissue inhibitor of metalloproteinase-2 (TIMP2) a known regulator of fibrosis. All had significantly higher expression in the lSSc-PAH group relative to healthy controls (p≤0.05, Figure 3). For IL1B, IL13Rα1, and TIMP2 there was a significant difference in expression between lSSc-PAH and lSSc-NoPAH samples (p≤0.05, Figure 3). The observed differences in gene expression between these groups are maintained even when excluding the four patients with extensive pulmonary fibrosis (two in each of the lSSc-PAH and lSSc-NoPAH groups), or the four patients with mildly elevated PCWP (>15 to ≤18, all in the lSSc-PAH group), indicating that these biomarkers of PAH are not primarily driven by pulmonary fibrosis or heart failure.

In addition, we carried out a complete secondary analysis excluding all patients with either PCWP >15 or extensive pulmonary fibrosis (both groups indicated in Figure 2). 305 probes were again selected using multi-class SAM and clustered hierarchically in the gene and sample dimensions. The dendrogram of patient clustering following this analysis is very similar to that seen when including these patients (Figure S3A) with the major groupings maintained. Therefore the inclusion of these patients has little effect on the overall results.

### Gene expression groups are associated with PAH severity

We examined the relationship between pulmonary severity metrics and the gene expression groups defined in the three-class analysis. The branch containing most of the lSSc-PAH patients is labeled ‘Group 1’, and the branch containing all healthy controls is labeled ‘Group 2’ (Figure 2A). Although SigClust suggested potential subgroups within Group 2, there was high variability at different p-values, suggesting more samples would be needed to define these groups conclusively. We have therefore considered these samples as a single group.

**Figure 4:**
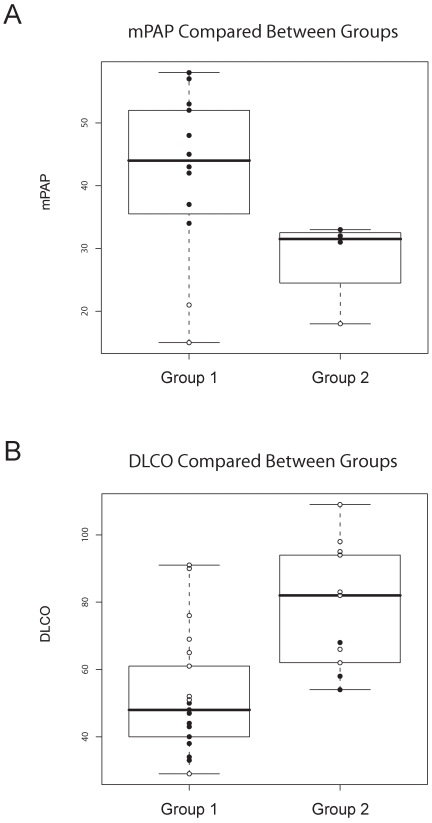
Distribution of PAH assessment measures between gene expression groups. ‘Group 1’ and ‘Group 2’ were defined by the major bifurcation in the clustering dendrogram of figure 2, with Group 1 containing all lSSc-PAH samples but three, and Group 2 containing all of the healthy control samples. Solid circles indicate PAH measures from lSSc-PAH patients, open circles indicate those from lSSc-NoPAH patients. A. mPAP measurements compared between patients in Group1 versus those in Group 2. B. DLCO values compared between Group 1 and Group 2.

Strikingly, when comparing the mean pulmonary arterial pressure (mPAP) values between Groups 1 and 2, the mPAP was higher for lSSc-PAH patients (Fig. 4A, solid black circles) in Group 1 versus Group 2. The mean PAP of the three patients with lSSc-PAH in Group 2 showed the lowest mPAPs of the patients with PAH, and these patients had a more intermediate gene expression compared to lSSc-PAH patients of Group 1. We also compared the diffusing capacity for carbon monoxide (DLCO) between the two groups, as isolated depression of diffusion capacity is associated with PAH in SSc [28]. DLCO was decreased in Group 1 for both SSc-PAH and SSc-NoPAH patients (Figure 4B, p-value = 0.000263). In a reciprocal pattern to that seen for the mPAPs, the three lSSc-PAH patients in Group 2 (solid black circles) had more preserved DLCO compared to the lSSc-PAH patients in Group 1. Therefore, lSSc-PAH patients in Group 2 show milder changes from normal in both mPAP and DLCO compared to lSSc-PAH patients of Group 1.

Three patients in Group 1 (2 lSSc-PAH: Pat22 and Pat86, and 1 lSSc-NoPAH: Pat89) and one patient in Group 2 (lSSc-PAH: Pat64) had extensive ILD. The difference in FVC between Group1 and Group 2 was not statistically significant, indicating that ILD was unlikely to be the primary clinical covariate driving gene expression in these groups (Table 1). Additionally, we were unable to detect clustering related to medications each patient was taking. Fifteen out of 36 patients were not on vasoactive medications at time of the first blood draw (Table 1). In patients taking vasoactive medications, the most common treatments were: phosphodiesterase type 5 (PDE5) inhibitors, sildenafil (4 lSSc-PAH and 1 lSSc-noPAH patient) and tadenafil (1 lSSc-PAH patient); epoprostenol (3 lSSc-PAH patients and 1 lSSC-NoPAH patient); and nifedipine (9 lSSc-NoPAH patients). Two patients, Pat60 and Pat35, started sildenafil before the second blood draw and showed similar gene expression between the two time points (Figure 2), indicating that this class of medication did not affect the expression analyses.

### Coordinate enrichment of biological processes

To investigate the pathways deregulated in lSSc with and without PAH, we performed a separate multiclass SAM analysis and selected a more inclusive list of 2,313 probes (FDR = 2.67%). This list was analyzed for enriched biological processes using the Database for Annotation, Visualization, and Integrated Discovery (DAVID). Figure S4 shows clusters of gene expression analyzed using DAVID. The gene expression signature found in all but three lSSc-PAH patients (Figure S4), showed enrichment for GO biological processes associated with proliferation and inflammatory responses (Benjamini-Hochberg corrected, p≤0.05), including *negative regulation of apoptosis* and *cell differentiation*, *I-kappaB kinase/NF-kappaB cascade*, *myeloid cell differentiation, response to external stimulus*, and *inflammatory response*. Genes included annexin A1 (ANAX1), chemokine ligand 2 (CCL2), BCL2 related protein A1 (BCL2A1), and tumor necrosis factor receptor a1 (TNFRSF1A).

### Gene expression associated with specific cell-types

To identify gene expression associated with specific cell types, as well as to seek out indications of cellular activation and differentiation, we used experimentally derived gene sets from isolated cells. Gene sets were obtained for B-cells, T-cells, macrophages, monocytes, immature and mature dendritic cells (DCs), and myeloid cells (DCs, macrophages, and monocytes)[29], [30]. While PBMCs do not contain macrophages, the gene expression profiles for these cells were included in the analysis due to the possibility of monocyte populations showing signs of differentiation into macrophages. We used Gene Set Enrichment Analysis (GSEA) to determine gene set signatures significantly overrepresented among the probes ranked by significance of differential expression between lSSc and healthy control samples. Four gene sets were significantly enriched at p≤0.1: myeloid cells (p = 0.013, FDR q-value = 0.070); dendritic cells (p = 0.014, FDR q-value = 0.056); macrophages (p = 0.011, FDR q-value = 0.059); and immature dendritic cells (p = 0.079, FDR q-value = 0.237). The monocyte gene set was fifth in the list ordered by significance and although above the p-value cutoff (p = 0.131), was below the suggested FDR cutoff of 0.25 (FDR q-value = 0.239). The enrichment of gene sets for the related cell types of myeloid, dendritic and macrophage cells, coupled with the enrichment of GO biological processes of *endocytosis* and *cell motility*, suggests the presence of monocytes undergoing cellular recruitment and differentiation into further cell types such as macrophages, particularly in lSSc-PAH patients. Gene expression and cellular differences in monocytes have been found between SSc and healthy control patients [31], [32], and the indication here of lSSc-PAH patients in particular having increased gene expression associated with macrophages and dendritic cells suggests an inflammation and injury response with cellular recruitment and differentiation in PAH patients. Figure S5 shows the GSEA enrichment plot and associated gene expression data for the top five gene sets.

### LSSc-PAH patients show elevated serum biomarkers of inflammation and vascular injury

**Figure 5:**
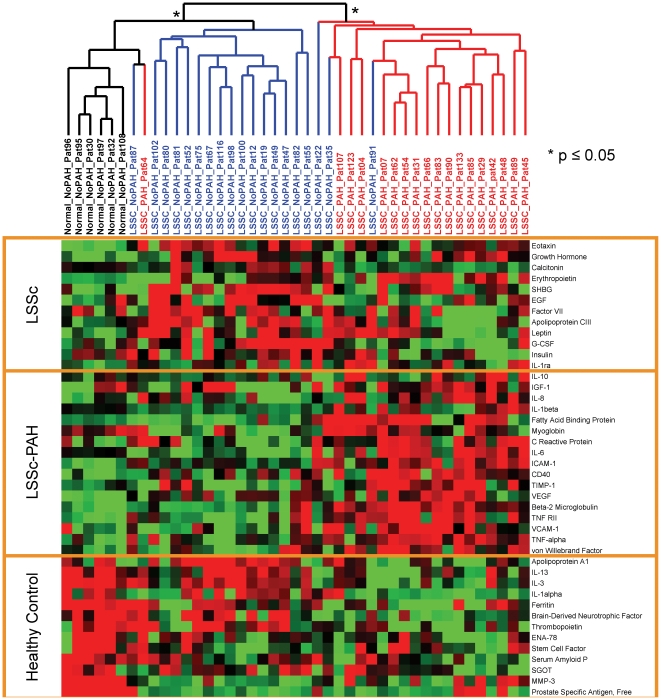
A subset of cytokines shows association with lSSc and lSSc-PAH. A MAP panel was used to profile the cytokines in plasma from lSSc-PAH, lSSc-NoPAH, and healthy control samples. Cytokines with significantly different levels between the three groups were selected using a multiclass SAM analysis (FDR<4.93%). 42 cytokines were selected and organized by 2 dimensional hierarchical clustering. The dendrogram shows lSSc-PAH (red), lSSc-NoPAH (blue), and healthy controls (black) group distinctly. Asterisks indicate stable groups as determined by SigClust. Increasing brightness of red indicates relative fold change increase in cytokine levels. Increasing brightness for green pixels indicates decreasing cytokine levels. Yellow boxes highlight the cytokines with increased levels in the three major groups, lSSc-PAH, lSSc regardless of PAH status, and healthy controls.

Serum samples were analyzed for 18 lSSc-PAH, 19 lSSc-NoPAH, and 6 healthy controls. Of these individuals, 13 lSSc-PAH, 18 lSSc-NoPAH, and 4 healthy controls were in the gene expression analysis. Levels of 89 cytokines were measured using Human Multi-Analyte Profiling (MAP) multiplexed immunoassays. To select cytokines with the greatest differential signal between the three groups, a multi-class SAM analysis was used to compare lSSc-NoPAH, lSSc-PAH, and healthy controls. The protein quantities for 42 cytokines (FDR = 4.93%) were clustered in patient and cytokine dimensions. Significantly stable clusters are indicated (p<0.05, Figure 5). LSSc-PAH, lSSc-NoPAH, and healthy controls clustered together except for lSSc-PAH-64 and lSSc-NoPAH-91 (Figure 5). Notably, lSSc-PAH-64 also had gene expression similar to the lSSc-NoPAH patients (Figure 2), confirming the gene expression findings.

Markers of vascular injury were increased in the lSSc-PAH patients, including Von Willebrand Factor (vWF) [33], C-reactive protein (CRP) [34], and vascular endothelial growth factor (VEGF). VEGF and vWF have been found upregulated in idiopathic pulmonary arterial patients [35]. Also found are fatty acid binding protein and myoglobin which are markers of acute myocardial infarction [36].

Found at higher levels in lSSc-PAH are cytokines characteristic of inflammatory response including: intercellular adhesion molecule 1 (ICAM-1), which was also increased in the microarray data and confirmed by qRT-PCR; vascular cell adhesion molecule 1 (VCAM-1); interleukin-8 (IL-8); interleukin-6 (IL-6); interleukin 1-beta (IL-1β); and tumor necrosis factor alpha (TNF-alpha). Consistent with the cytokine data, TNF-alpha inducible protein 2 (TNFAIP2) shows increased gene expression in the lSSc-PAH patients. Increased levels of tumor necrosis factor receptor superfamily member 1B (TNFRII/TNFR1B) [37] and Beta2–microglobulin (β2m) are also observed in the lSSC-PAH patients. High levels of circulating TNFRII and β2m have been reported in SSc [31], [38], however these biomarkers have not previously been investigated specifically for lSSc-PAH patients.

## Discussion

Our results demonstrate gene expression differences between the majority of lSSc patients and healthy controls, as well as differences between lSSc-PAH and lSSc-NoPAH patients. PBMC gene expression divides lSSc patients based on the presence and severity of PAH as assessed by PAP and DLCO. Although some patients with PAH fell into the group without PAH in the hierarchical clustering analysis, these patients uniformly had the mildest PAH in the study. Further studies will be required to determine if the lSSc patients without PAH but with gene expression more similar to patients with PAH, are at higher risk of eventually developing this complication. Our current data show the gene expression of lSSc-PAH and lSSc-NoPAH samples are stable over the course of approximately one year. Further analyses over an extended period are needed to rigorously test whether gene expression can predict the onset of PAH, and how gene expression in lSSc patients changes over time.

In addition to gene expression differences between lSSc-PAH and lSSc-NoPAH patients, we found differences in cytokines between the two groups. The cytokine and gene expression profiles suggest activated and differentiating monocytes. Notably, IL1-beta, produced primarily by stimulated monocytes [39], and Caspase 1, an important enzyme for cleaving the IL-1beta precursor [40] were both increased in lSSc-PAH. Gene expression of TLR4, a key receptor for monocyte activation and IL1 upregulation, and MYD88, a TLR signaling molecule, was also upregulated. Consistent with TLR activation of NF-kappa B [41], GO biological processes enriched in lSSc-PAH include the *I-kappaB kinase/NF-kappaB cascade*. Collectively these observations suggest that activated monocytes play an important role in the inflammatory response in lSSc-PAH patients.

The combined gene expression and cytokine data indicate the involvement of other myeloid cell types beyond monocytes in lSSc-PAH patients. The most significant GSEA results were for gene sets associated with myeloid cells, dendritic cells, and macrophages. GO biological processes of *myeloid cell differentiation*, *endocytosis*, *cell motility*, and *cell projection biogenesis* were also enriched. Dendritic cells have been implicated in the immunopathology of IPAH with increases in DCs found in vasculopathy rat models of PAH, and increased DC infiltrates in affected vessels in human IPAH [42]. lSSc-PAH patients showed increased IL-6, which signals monocytes to differentiate into macrophages, has previously been shown upregulated in PAH patients and can induce PAH in transgenic mice [43], [44], [45]. Cytokines ICAM-1 and VCAM-1 are increased in lSSc-PAH, along with increased expression of the ICAM-1 gene. These cytokines are induced through IL1B and TNF-alpha (both increased in lSSc-PAH patients on the cytokine array) and initiate the binding of monocytes to the endothelium. Through a combined gene expression and cytokine analysis approach, these data support a role for activated DCs and macrophages in lSSc-PAH. LSSc-PAH patients also showed upregulated circulating levels of other inflammatory cytokines, IL1B and TNF-alpha and downstream targets of these mediators, notably the adhesion molecules, ICAM-1 and VCAM-1 These data support a role for activated DCs and macrophages in lSSc-PAH.

Grigoryev et al. found genes upregulated in both IPAH and SSc-PAH compared to healthy controls, as well as a relationship between decreasing gene expression and increasing right atrium pressure [14]. In this study we found upregulation of some of the same genes including adrenomedulin (ADM) and pentraxin-related gene (PTX3), but we found increased expression of these genes in some of the lSSc-NoPAH patients as well.

We found increases in some of the same cytokines, including ICAM-1, TIMP1, and vWF, previously noted by Duan et al. to be increased in lSSc patients compared to healthy controls [31]. We found levels of these cytokines were higher in lSSC patients with PAH compared to those without PAH, indicating the importance of these as biomarkers of lSSc-PAH.

Increased expression of inflammatory cytokines in the serum of SSc patients with PAH suggests the possibility that these cytokines might play a role in pathogenesis. Notably, TNF was found to be increased in the serum of lSSc-PAH patients in this study and TNF increases pulmonary vascular resistance [46], stimulates endothelin-1 [47] and leads to PAH in TNF transgenic mice [48] TNF and TNF-regulated genes are increased in patients with rheumatoid arthritis, where TNF inhibition provides a clear therapeutic benefit [49]. In SSc, elevated TNF has been described in diffuse cutaneous patients with pulmonary fibrosis [50]. One recent open label study has suggested that TNF inhibition may ameliorate skin disease [51] and a case report has suggested some possible value for PAH [52]. IL6 was found to be increased in lSSc-PAH patients on the cytokine array, and IL6 is also associated with PAH in the context of chronic obstructive pulmonary disease (COPD) [53]. Further supporting a possible role in SSc-associated PAH, IL6 transgenic mice develop PAH [45], and hypoxia-induced PAH in mice is ameliorated in IL6-deleted mice [54]. In our study, although IL6 protein was elevated in the serum, IL6 mRNA levels were not elevated in PBMC samples, suggesting that IL6 might be secreted primarily from other cell types, such as endothelial cells, fibroblasts or neutrophils. Thus these inflammatory mediators, possibly through activation of an innate immune response, may play a role in SSc-associated PAH.

Our results provide potential biomarkers that identify patients with lSSc, showing a specific subset of lSSc patients with PAH that can be identified through cellular and circulating biomarkers, and suggest pathogenic cellular and immunologic pathways that are upregulated in these patients. The gene expression profiles in PBMCs in our study may also provide biomarkers to predict the risk of lSSc patients for developing PAH.

## Methods

### Ethics Statement

This study was approved by the Boston University Medical Center Institutional Review Board, and the Committee for the Protection of Human Subjects at Dartmouth College. All patients signed informed written consent forms approved by the Boston University Medical Center Institutional Review Board.

### Patient Selection

Subjects included limited cutaneous systemic sclerosis (lSSc) patients according to criteria in LeRoy et al. [55] and healthy controls. Subjects with lSSc were stratified into those with or without PAH on the basis of echocardiogram and pulmonary artery catheterization according to consensus criteria with exceptions noted below [56]. LSSc subjects with echocardiogram showing a systolic pulmonary arterial pressure (PAP) <35 mm Hg and no clinical features suggesting PAH were considered to not have PAH. Subjects showing evidence of PAH by echocardiogram or other clinical features who underwent right heart catheterization and showed a mean PAP≤25 mm Hg were also considered to not have PAH (4 patients). Subjects showing evidence of PAH by echocardiogram or other clinical criteria who underwent catheterization and showed mean PAP >25 mm Hg and pulmonary capillary wedge pressure (PCWP) ≤15 were considered to have PAH, or with PCWP >15, but ≤18 considered to have PAH if adjudicated by the attending pulmonologist on the basis of PVR, PAd-PCWP gradient and transpulmonary gradient (see further details in results section for individual patients meeting this criteria). Most subjects entered into the study had no or minimal interstitial lung disease. The extent of disease in those with interstitial lung disease (9 subjects) was stratified as either mild (5 subjects) or extensive (4 subjects) according to previously described methodology using high-resolution chest computerized tomography (HRCT) and forced vital capacity (FVC) criteria [57]. Blood was collected from patients on the day of catheterization for catheterized subjects, or within three months of the date of echocardiogram for subjects who were not catheterized.

### Peripheral Blood Mononuclear Cell Isolation

PBMCs were collected in Becton Dickinson vacutainer CPT tubes and processed within 30 minutes after collection. Tubes were centrifuged at 1800×g for 30 min at room temperature (18–25°C). The PBMC cell layer was then transferred to a 15 mL tube, and the PBMCs washed twice with PBS and lysed in RNeasy RLT buffer (Qiagen, Valencia, CA).

### RNA isolation and microarray hybridization

Total RNA was prepared from PBMCs using the RNeasy Mini Kit (Qiagen). 250 ng of RNA was converted to cDNA and amplified as labeled cRNA using a Low RNA Input Fluorescent Linear Amplification Kit (Agilent Technologies). Patient and healthy control RNA were labeled with Cy3 fluorescent dye, and Universal Human Reference RNA (Stratagene) was labeled with Cy5 fluorescent dye. Labeled cRNA was hybridized to Agilent 4×44,000 element DNA microarrays in a reference-based design as previously described [10], with the following changes. Patient or healthy control cRNA was co-hybridized with UHR cRNA to microarrays for 17 hours at 65°C. Arrays were washed for 1 minute each in 6× SSPE, 0.005% N-Lauroylsarcosine at room temperature and then in 0.06× SSPE, 0.005% N-Lauroylsarcosine at 37°C. This was followed by an acetonitrile wash for 1 minute at room temperature and Stabilization and Drying Solution for 30 seconds at room temperature. Microarrays were scanned, processed, and data normalized and filtered as previously described [10].

### Data Access

All microarray data from this study has been deposited to NCBI's Gene Expression Omnibus (GEO; http://www.ncbi.nlm.nih.gov/geo/; Accession Number GSE19617) and is MIAME compliant.

### Gene Selection and Hierarchical Clustering

Gene selection was performed using Significance Analysis of Microarrays (SAM) [17]. For the two class unpaired t-test, arrays were grouped by lSSc vs. healthy controls and probes selected with a false discovery rate (FDR) <0.18%. The gene expression levels of the X (inactive)-specific transcript (XIST) gene were removed from the analysis because it caused one healthy control sample to group by gender rather than disease (Figure S6).

For the multiclass analysis, samples were divided by lSSc-PAH, lSSc-NoPAH, and healthy controls. We performed a stringent analysis that identified 305 probes (FDR<0.14%) and a less stringent analysis that identified 2,313 probes (FDR<2.67%) (Figure S5). Average linkage hierarchical clustering was performed as previously described [10].

### Pathway Analysis

The Database for Annotation, Visualization, and Integrated Discovery tool (DAVID) [58] was used to analyze coordinately regulated groups of genes for enriched GO biological processes.

Gene Set Enrichment Analysis (GSEA) [59], was used to determine enrichment of cell-type specific gene sets. Genes were ranked by significance of differential expression between lSSc and healthy controls. For each gene set, GSEA determined if the set was found at the top or bottom of the ranked list. Significance was determined by permuting the data. Gene sets for B and T cells were obtained from Palmer et al. [29]. Gene sets for macrophages, monocytes, immature and mature dendritic cells, CD3/CD28 activated T cells, total PBMCs and myeloid cells (DCs, macrophages, monocytes) were obtained from Haider et al. [30].

### Quantitative RT-PCR

RT-PCR was carried out as previously described [60], using ABI primers for the following genes: ICAM1 (Hs00164932_m1), IFNγR1 (Hs00166223_m1), IL1B (Hs01555410_m1), IL13Rα1 (Hs00609817_m1), JAK2 (Hs01078136_m1), AIF1 (Hs00357551_g1), CCR1 (Hs00174298_m1), ALAS2 (Hs00163601_m1), TIMP2 (Hs00234278_m1). Relative RNA quantity was determined using the delta-delta CT method [61].

### Cytokine Panel

Serum samples were collected from 43 patients, and sent to Rules-Based Medicine (http://www.rulesbasedmedicine.com) for the Human Multi-Analyte Profiling (MAP) multiplexed immuno assay. MAP assays were carried out two different times including approximately equal numbers of samples from all three subject groups. The first assay obtained analyte measurements for 89 cytokines, the second for 90 cytokines. Results that were not detectable were replaced with the reported least detectable amount for that cytokine. To control for bias between the two MAP assays, the data in the second MAP assay were normalized for each cytokine to the proportion of the average values in the first MAP assay divided by the average values in the second MAP assay. Multiclass SAM analysis was used to compare cytokines detected between LSSc-NoPAH, LSSc-PAH, and healthy controls, and the resultant data at an FDR cutoff of 4.93% was clustered in both the patient and cytokine dimension.

### Statistical Analysis

The R statistical package was used for box plots, two-group t-test analysis of FVC, DLCO, and PAP, as well as log transformation, ANOVA, and Tukey H.S.D. analysis of the qRT-PCR data.

### SigClust

An iterative implementation in R of SigClust [18] was used to determine the number of stable clusters of arrays found after the hierarchical clustering of the gene expression data as described in [10]. Three P-value cutoffs of p≤0.05, p≤0.01, and p≤0.001 were used for SigClust analysis for each dataset.

## Supporting Information

Figure S1 All gene names for Figure 1. This file is intended to be viewed digitally, as the text is small and requires the ability to zoom in and out.
(0.80 MB PDF)

Figure S2 All gene names for Figure 2. This file is intended to be viewed digitally, as the text is small and requires the ability to zoom in and out.
(1.07 MB PDF)

Figure S3 Comparison of three group clustering in the presence and absence of a patients with elevated wedge pressure or extensive ILD. A. Patients with PCWP >15 (mm Hg) and those with extensive ILD were removed and the analysis shown in Figure 2 repeated. The hierarchical clustering dendrogram was generated using 305 probes selected by a multi-group SAM analysis. Even without these patients, the majority of the arrays that fell within “group 1” (purple) in the previous analysis, still were in group 1, and likewise for those arrays that fell within “group 2” (black) in the previous analysis. B. This shows the same dendrogram as that of S3A, in this case the arrays are indicated in color according to diagnosis, lSSc-PAH (red), lSSc-NoPAH (blue), healthy controls (green).
(0.17 MB PDF)

Figure S4 Gene expression differentiating lSSc-PAH, LSSc-noPAH and healthy controls, less stringent cutoff. Supplemental figures 3A and B show the clustering dendrogram and resultant heatmap after hierarchically clustering in the array and gene dimension the resultant 2313 probes that passed a multiclass SAM analysis with an FDR of 2.67%. Figure S2B shows the clustering dendrogram with the sample identifiers. A black bar beneath the sample identifiers connects technical replicates, and samples collected approximately one year later samples are connected to the baseline samples by a yellow line. To seek out pathways associated with coordinate gene expression utilizing David analysis, gene lists were created from the regions marked in red, green, purple, and blue.
(0.76 MB TIF)

Figure S5 Gene Set Enrichment Analysis (GSEA) for different cell type signatures in the gene expression of lSSc-PAH, lSSc-NoPAH and healthy controls. Gene expression signatures for myeloid cells (A), monocytes (B), macrophages (C), IDCs (D), and DCs (E) from Haider et al. were found to be enriched in the gene expression profiles using GSEA. The top panel shows the GSEA enrichment plot. The bottom panel shows the gene expression plot from the PBMC dataset for genes/probes that matched each respective gene list (the gene names associated with each probe are available in supplemental data). Gene expression in blue represents decreased gene expression, while red represents increased gene expression. The Normalized Enrichment Score (NES) is shown for each gene set along with the FDR q-value. An FDR q-value<0.25 is considered to be significant. The lSSc-PAH samples consistently show increased expression (boxed, red cells) relative to the lSSc-noPAH samples and healthy controls (highlighted in yellow) for each gene set. This suggests increased expression of genes associated with these cell types in the lSSc-PAH samples. The most significant enrichment was found in the myeloid cell gene set, whereas the least significant enrichment was found in the monocyte gene set.
(4.51 MB TIF)

Figure S6 Disease vs. No Disease SAM Analysis with Additional Probe. A. Clustering dendrogram for the hierarchical clustering in the array and gene dimension of the 54 arrays and the 207 probes that most distinguished via gene expression between lSSc and healthy control samples (FDR cutoff<0.18%), including X (inactive)-specific transcript (XIST), a gene expressed exclusively from the X inactivation center of the inactive X chromosome. The sample identifiers are marked with lSSc in red and healthy control in black. A black bar beneath the sample identifiers connects technical replicates. Samples collected approximately one year apart are connected to baseline samples by a yellow line. The dendrogram tree is marked to indicate if samples were from lSSc patients, or healthy control. B. Heat map showing gene expression after hierarchical clustering in the array and probe dimension. Red is upregulation of gene expression, green is down regulation of gene expression.
(0.75 MB TIF)

Data File S1 This file list the 206 probes shown in Figure 1 along with the gene expression matrix. Genes were selected with a false discovery rate (FDR) <0.18% hierarchically clustered by both sample and probe, for the genes most consistently and significantly differentially expressed between lSSc patients (lSSc-NoPAH and lSSc-PAH) and healthy control.
(0.25 MB XLS)

Data File S2 This file contains the complete list of 305 probes and data matrix for Figure 2. Genes were selected with an FDR<0.14%, hierarchically clustered in both the gene and sample dimension, for the gene expression differences that distinguish lSSc-PAH, lSSc-NoPAH, and healthy control samples.
(0.34 MB XLS)
