# Supplementary figures and images for: Limited Systemic Sclerosis Patients with Pulmonary Arterial Hypertension Show Biomarkers of Inflammation and Vascular Injury
Source: PLoS One. 2010 Aug 17;5(8):e12106. doi: 10.1371/journal.pone.0012106 (PMC2923145; doi:10.1371/journal.pone.0012106)

Figure S1

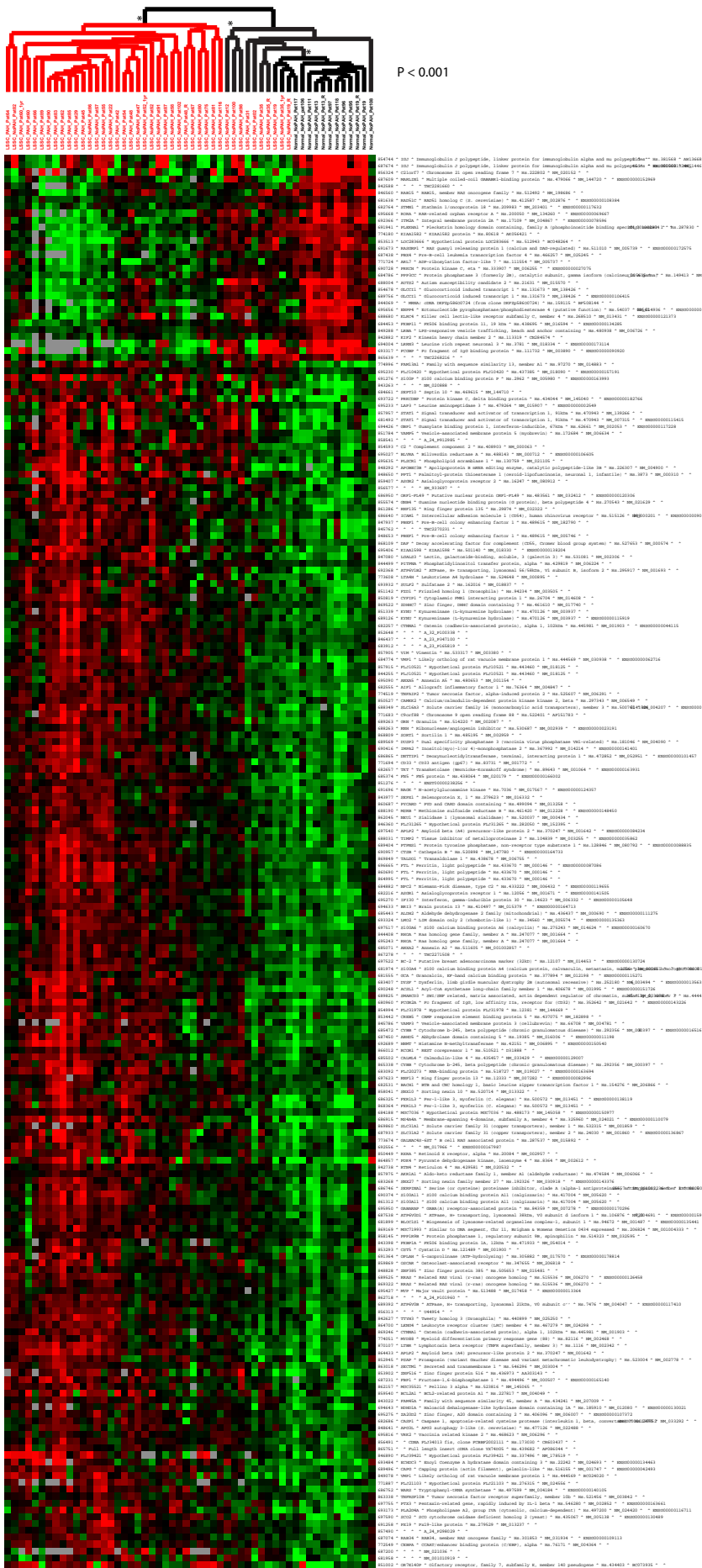

Supplement: Figure S1 — All gene names for Figure 1. This file is intended to be viewed digitally, as the text is small and requires the ability to zoom in and out. (0.80 MB PDF) [file pone.0012106.s001.pdf]

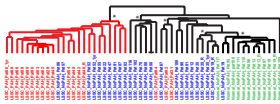

\*P < 0.001

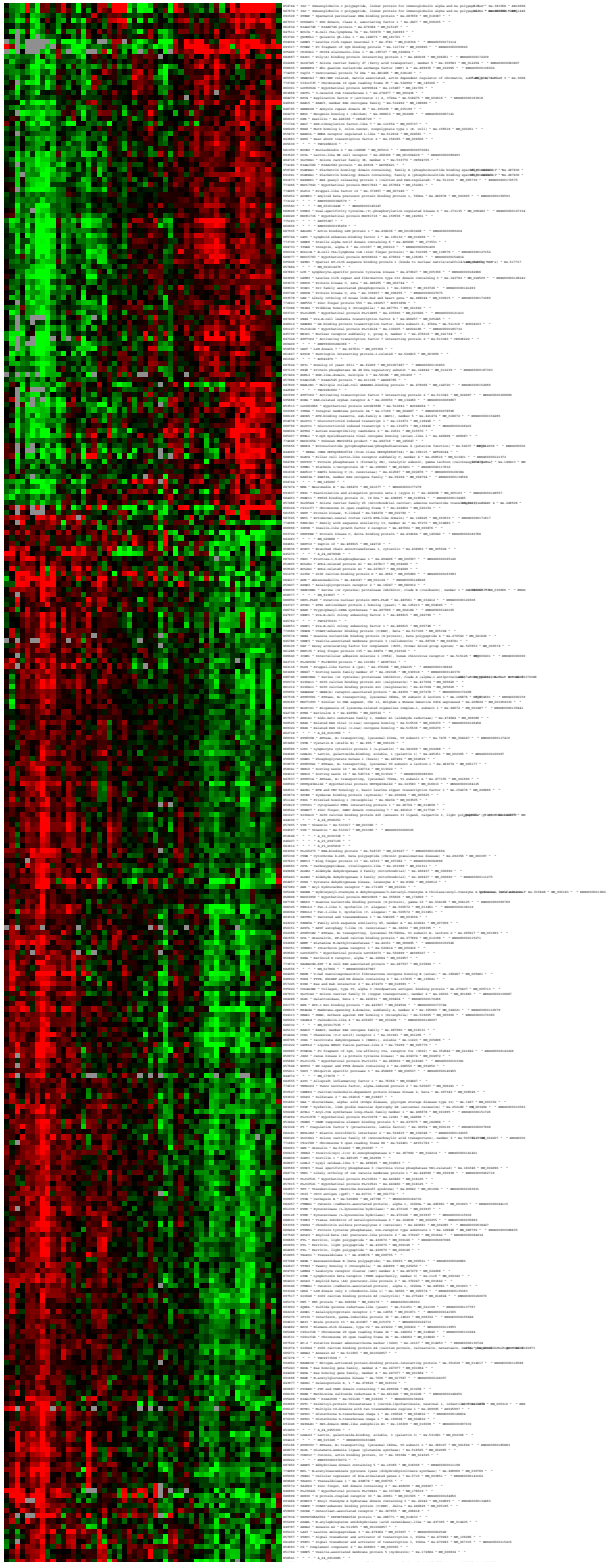

Supplement: Figure S2 — All gene names for Figure 2. This file is intended to be viewed digitally, as the text is small and requires the ability to zoom in and out. (1.07 MB PDF) [file pone.0012106.s002.pdf]

Figure S3

A

Dropping out Extreme ILD and PCWP > 15

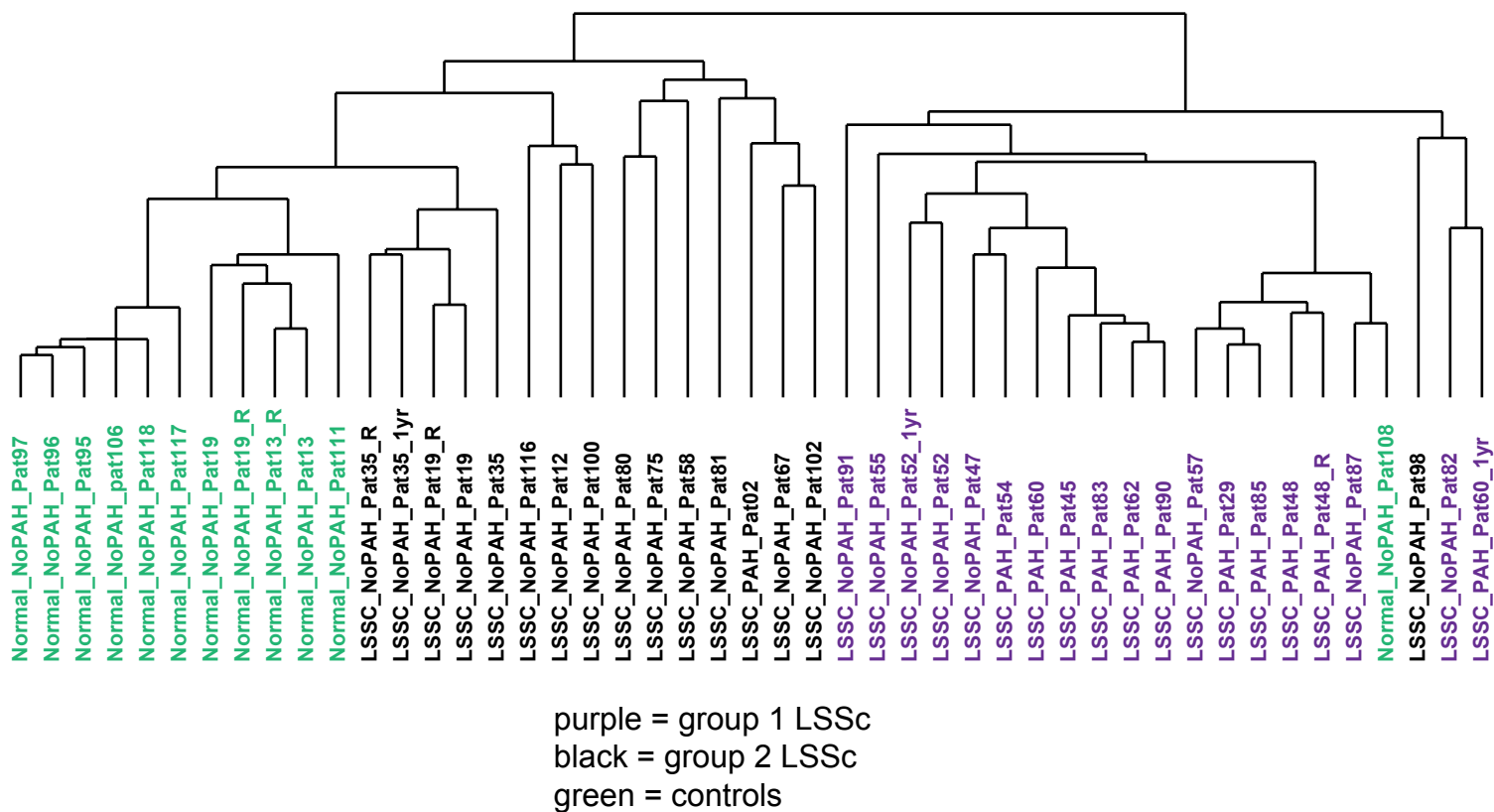

B

Dropping out Extreme ILD and PCWP > 15

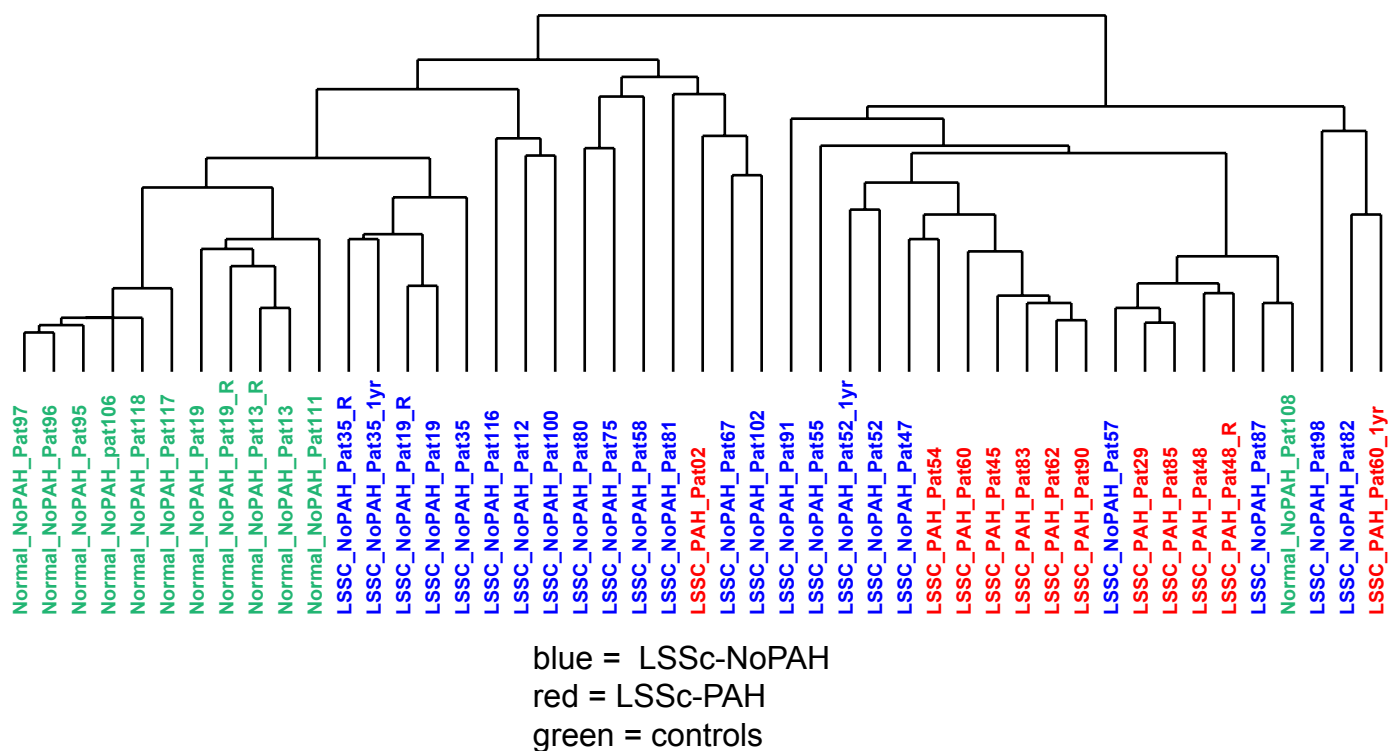

Supplement: Figure S3 — Comparison of three group clustering in the presence and absence of a patients with elevated wedge pressure or extensive ILD. A. Patients with PCWP >15 (mm Hg) and those with extensive ILD were removed and the analysis shown in Figure 2 repeated. The hierarchical clustering dendrogram was generated using 305 probes selected by a multi-group SAM analysis. Even without these patients, the majority of the arrays that fell within “group 1” (purple) in the previous analysis, still were in group 1, and likewise for those arrays that fell within “group 2” (black) in the previous analysis. B. This shows the same dendrogram as that of S3A, in this case the arrays are indicated in color according to diagnosis, lSSc-PAH (red), lSSc-NoPAH (blue), healthy controls (green). (0.17 MB PDF) [file pone.0012106.s003.pdf]

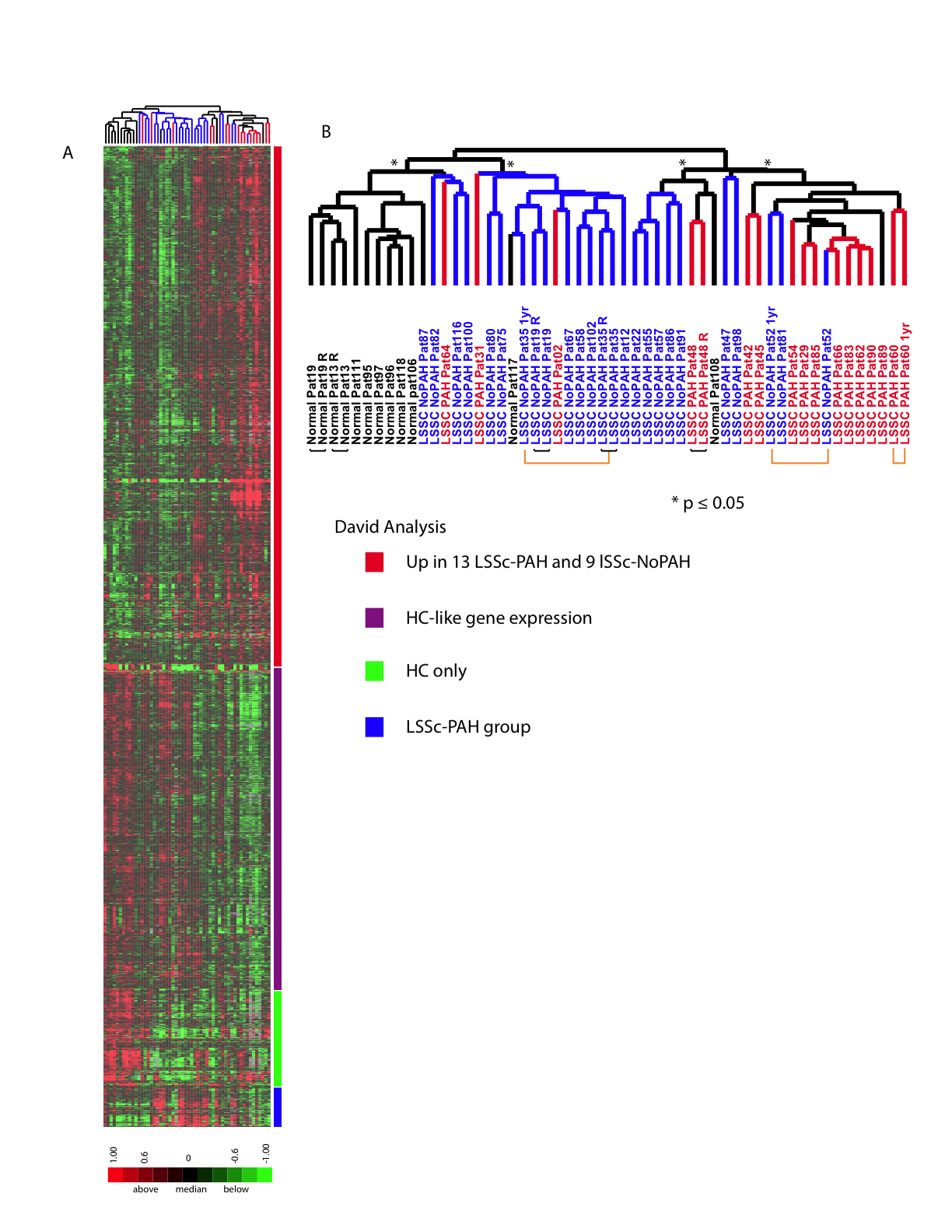

Supplement: Figure S4 — Gene expression differentiating lSSc-PAH, LSSc-noPAH and healthy controls, less stringent cutoff. Supplemental figures 3A and B show the clustering dendrogram and resultant heatmap after hierarchically clustering in the array and gene dimension the resultant 2313 probes that passed a multiclass SAM analysis with an FDR of 2.67%. Figure S2B shows the clustering dendrogram with the sample identifiers. A black bar beneath the sample identifiers connects technical replicates, and samples collected approximately one year later samples are connected to the baseline samples by a yellow line. To seek out pathways associated with coordinate gene expression utilizing David analysis, gene lists were created from the regions marked in red, green, purple, and blue. (0.76 MB TIF) [file pone.0012106.s004.tif]

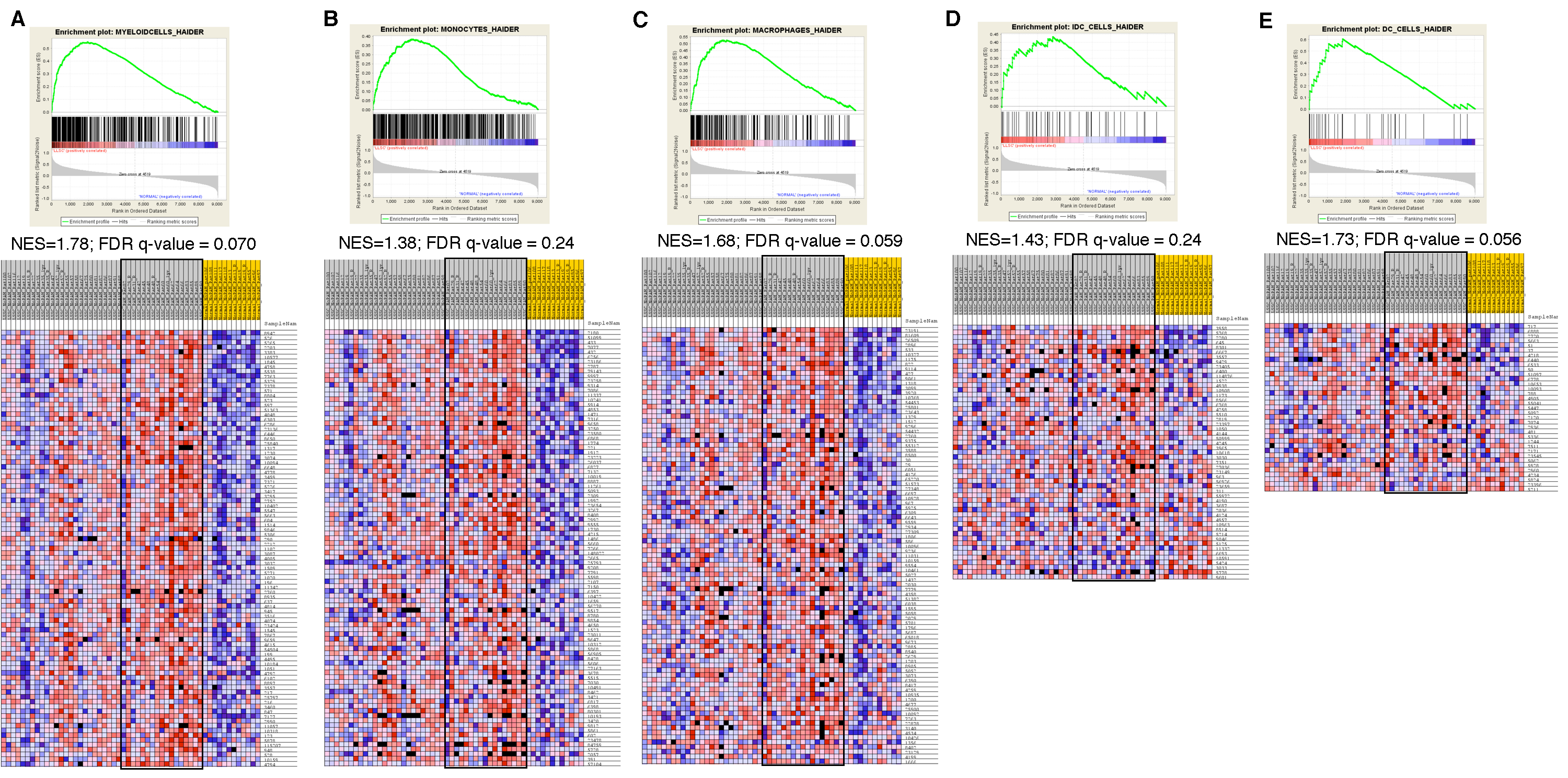

Supplement: Figure S5 — Gene Set Enrichment Analysis (GSEA) for different cell type signatures in the gene expression of lSSc-PAH, lSSc-NoPAH and healthy controls. Gene expression signatures for myeloid cells (A), monocytes (B), macrophages (C), IDCs (D), and DCs (E) from Haider et al. were found to be enriched in the gene expression profiles using GSEA. The top panel shows the GSEA enrichment plot. The bottom panel shows the gene expression plot from the PBMC dataset for genes/probes that matched each respective gene list (the gene names associated with each probe are available in supplemental data). Gene expression in blue represents decreased gene expression, while red represents increased gene expression. The Normalized Enrichment Score (NES) is shown for each gene set along with the FDR q-value. An FDR q-value<0.25 is considered to be significant. The lSSc-PAH samples consistently show increased expression (boxed, red cells) relative to the lSSc-noPAH samples and healthy controls (highlighted in yellow) for each gene set. This suggests increased expression of genes associated with these cell types in the lSSc-PAH samples. The most significant enrichment was found in the myeloid cell gene set, whereas the least significant enrichment was found in the monocyte gene set. (4.51 MB TIF) [file pone.0012106.s005.tif]

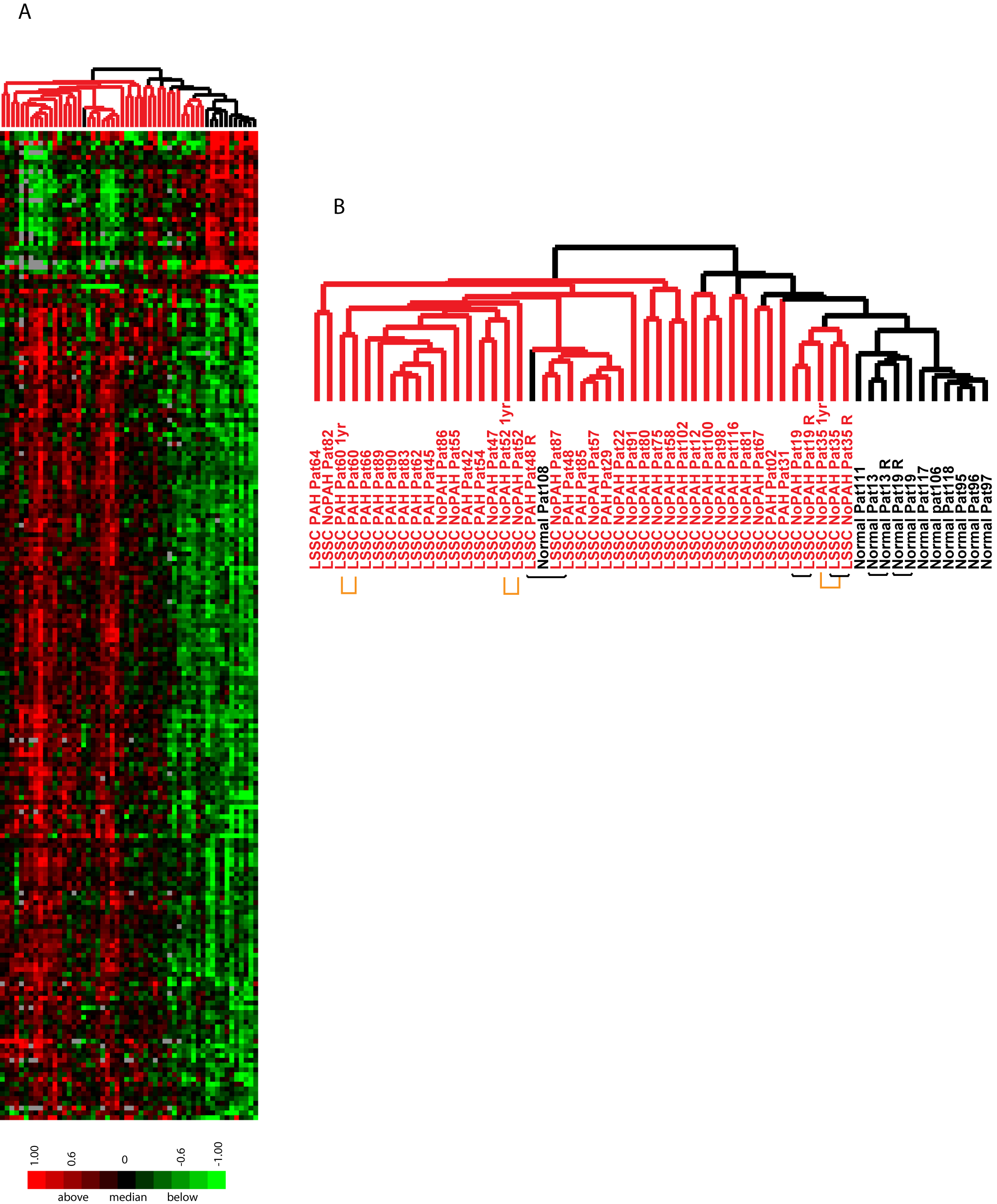

Supplement: Figure S6 — Disease vs. No Disease SAM Analysis with Additional Probe. A. Clustering dendrogram for the hierarchical clustering in the array and gene dimension of the 54 arrays and the 207 probes that most distinguished via gene expression between lSSc and healthy control samples (FDR cutoff<0.18%), including X (inactive)-specific transcript (XIST), a gene expressed exclusively from the X inactivation center of the inactive X chromosome. The sample identifiers are marked with lSSc in red and healthy control in black. A black bar beneath the sample identifiers connects technical replicates. Samples collected approximately one year apart are connected to baseline samples by a yellow line. The dendrogram tree is marked to indicate if samples were from lSSc patients, or healthy control. B. Heat map showing gene expression after hierarchical clustering in the array and probe dimension. Red is upregulation of gene expression, green is down regulation of gene expression. (0.75 MB TIF) [file pone.0012106.s006.tif]
